# Recent Insights into the Morphological, Nutritional and Phytochemical Properties of Indian Gooseberry (*Phyllanthus emblica*) for the Development of Functional Foods

**DOI:** 10.3390/plants13050574

**Published:** 2024-02-20

**Authors:** Pawar Gayatri Avinash, Hamid, Rafeeya Shams, Kshirod Kumar Dash, Ayaz Mukarram Shaikh, Diána Ungai, Endre Harsányi, Tejas Suthar, Béla Kovács

**Affiliations:** 1 Department of Food Technology and Nutrition, Lovely Professional University, Phagwara 144001, Indiahamidfst6789@gmail.com (H.);; 2 Department of Food Processing Technology, Ghani Khan Choudhury Institute of Engineering and Technology Malda, Malda 732141, India; 3 Faculty of Agriculture, Food Science and Environmental Management, Institute of Food Science, University of Debrecen, 4032 Debrecen, Hungary; 4 Agricultural Research Institutes and Academic Farming (AKIT), Faculty of Agriculture, Food Science and Environmental Management, University of Debrecen, 4032 Debrecen, Hungary; 5 Independent Researcher, Chicago, IL 60616, USA

**Keywords:** Indian gooseberry, nutritional value, phytochemical properties, antioxidants, clinical studies

## Abstract

Aonla, commonly known as Indian gooseberry (*Phyllanthus emblica*), is a plant native to India with various therapeutic and dietary benefits. This review covers the taxonomical, morphological, and species-level classifications of aonla fruit, including its flower biology, maturation, harvesting, and yield metrics. It also discusses the nutritional, physico-chemical, and phytochemical characteristics and the total antioxidant and antimicrobial activities and mineral compositions of several aonla fruit cultivars. Additionally, the health benefits of aonla are reviewed, including its analgesic, antipyretic, antioxidative, anti-inflammatory, anti-aging, ulcerogenic, chemo-protective, neuroprotective, free radical scavenging, hypoglycaemic, and immunogenic properties, which make it beneficial in the treatment and prevention of various illnesses. Further various forms of fruit extract are also considered to be beneficial for the improvement of plant and animal health. Overall, aonla is a valuable fruit with significant potential for use in improving human health.

## 1. Introduction

Aonla, also known as the Indian gooseberry (*Emblicaofficinalis*, Gaertn. syn. *Phyllanthusemblica* Linn.), is a fruit native to India and is highly valued for its medicinal and nutritional properties. It is a member of the Euphorbiaceae family, and it is referred to as a wonder fruit for health [1]. It is indigenous to India, Shri-Lanka, Cuba, Thailand, Japan, Malaysia, and China [2]. Andhra Pradesh, Bihar, Gujarat, Haryana, Karnataka, Maharashtra, Madhya Pradesh, Rajasthan, Uttarakhand, Uttar Pradesh, Tamil Nadu, and West Bengal are among the states where it is commercially grown [3]. Aonla is produced yearly in 1046 thousand MT (millions of tons) on an area of 92,000 hectares that is cultivated across the nation [4].

Aonla fruit is considered the richest source of vitamin C due to its extraordinarily high vitamin C content in comparison to other commonly existing fruits [5]. Aonla fruit is also rich in proteins, fibres, and carbohydrates and is a good source of minerals like calcium, phosphorous, and iron [6]. Due to these compounds, it has strong antiviral, cardiotonic, and hyperglycaemic properties, which give it significant medical value. It is extensively utilized in both unani and ayurvedic therapy [7]. Aonla is used to manufacture ayurvedic medicines such as ashokarishta, chavanprash, triphalamasin, and triphala which are traditional specific formulations made by utilizing aonla fruit or with its powder [8]. Aonla has several therapeutic characteristics, and its many formulations and parts are used to cure a variety of illnesses. For example, fresh or dried fruits are used to treat diarrhoea, jaundice, and inflammation [9]. Fruit is used to cure several illnesses, including asthma, anaemia, bronchitis, cancer, the common cold, diabetes, loss of hair, greying of hair, scurvy, stress, and memory deterioration [10]. Aonla is highly acidic in nature, and it also has an astringent taste, due to which it is generally not consumed as a fresh fruit, but its processed forms have great potential for utilization [11]. Aonla powder, drinks, confectionery, juice, pickles, preserves, syrup, etc., are value-added products that are commercially available [8].

Aonla contains vitamin A, which is responsible for reducing age-related muscular degeneration. Total soluble fibres of aonla help in decreasing the risk of irritable bowel syndrome and help in maintaining blood sugar levels and bowel motions. Amla controls the lens protein aggregation and insolubility that arise from hyperglycaemia [12]. Amla has anticancer potential since it contains high-polyphenolic components. Polyphenols play an important role in anti-inflammatory and anti-carcinogenic activities [13]. The human body produces the neurotransmitter norepinephrine with the help of a large amount of vitamin C, which improves brain function in dementia patients. It is also employed in manufacturing cosmetics for anti-aging and is great for skin care [14]. For patients with diabetes, the amla fruit is used as a preventative measure against the development of neuropathy [15]. Aonla has a variety of pharmacological properties, including those that are anti-inflammatory, analgesic, and antioxidant properties. This fruit is used as a strong rasayana in ayurveda to cure a wide range of illnesses, including inflammation, jaundice, diarrhoea, and many more [10].

## 2. Species Classification

*Emblica officinalis* is widely cultivated in India. It includes other closely related species, such as *Emlica myrobalan*, which contains medium-sized to relatively small foliage. *Emblica fischeri* is a species used to produce pickles, and *Phyllanthus indofischeri* is a wide crown tree that may be found in south Indian scrub forests. These species are largely grown for aesthetic purpose [16].

### 2.1. Flower Biology of Aonla

**Figure 1:**
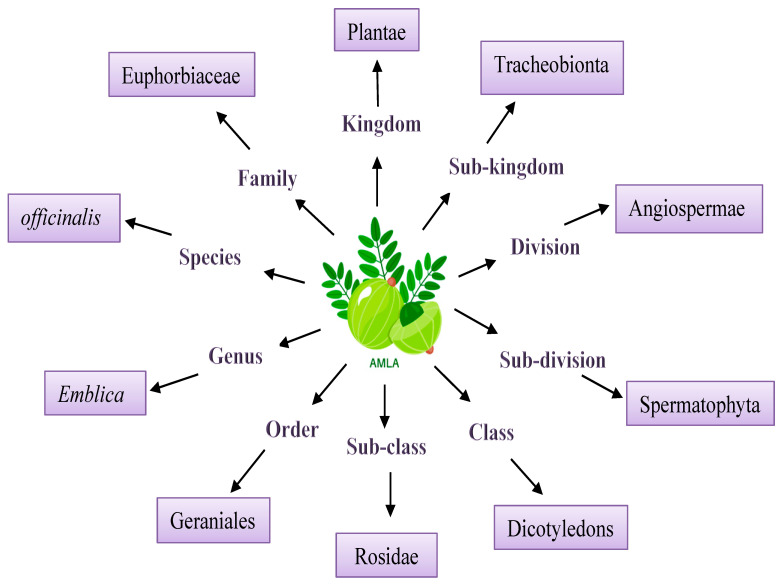
Taxonomical classification of Aonla.

The Indian gooseberry has a somatic chromosomal number of 2n = 28, despite having a 2n = 26 basic chromosome number [16]. Flowers are held on fixed stems as axillary cymules in the axils of the leaves. There are male flowers in the basal cymules, followed by central female bloom (occasionally two) and numerous lateral male flowers at each node [17]. Either an androecium or gynoecium composes a typical flower of aonla. Although there might be between 5 and 7 perianth segments, the most common number is usually 6. It has a male-to-female ratios of 307:1 and 197:1, indicating a significant variation in sex expression. The stigma begins to respond on the third day of anthesis and continues to be responsive for a further 48 h, and timing with the female flowers is staged 72 h after opening [16]. The taxonomical classification of aonla is presented in Figure 1 [18,19].

### 2.2. Morphological Characteristics of Different Aonla Cultivars

Various cultivars of aonla like Chakaiya, Banarasi, Francis, Kanchan, Krishna, Anand-2, Anand-1, NA-10, and NA-7 and their morphological characteristics, including respective features, tree height, tree habit, tree form, leaf size, leaf form, leaf width, leaf apex, foliage, and inflorescence colour, have been described. The tree height and habit varied among the cultivars, with Anand-2 and Anand-1 observed to have tall upright trees, NA-7 having tall spreading trees, Francis having tall drooping trees, and Kanchan having tall semi-spreading trees [20]. The smallest plants were observed in Chakaiya and Krishna, while the tallest plants were observed in Anand-2 and Anand-1 [21]. Foliage colour was observed to be grey in Chakaiya, Banarasi, Kanchan, Krishna, Francis, Anand-2, and Anand-1, while Kanchan, NA-10, and NA-7 had whitish/grey tree trunks [1]. The inflorescence colour varied among the cultivars, with Banarasi and Krishna having deep pink inflorescences; Francis having yellowish-green inflorescences; Chakaiya and Kanchan having pinkish-green inflorescences; Anand-1, NA-7, and NA-10 having green to light pink inflorescences; and Anand-2 had light green to pinkish inflorescences [21]. The leaf shape was observed to be oblong in Chakaiya, Banarasi, Krishna, NA-10, Anand-2, and Anand-1; oval oblong in Francis and Kanchan; and elliptical in NA-7. The two primary types of leaf apex were acute and obtuse, with all varieties except Chakaiya and Kanchan possessing a blunt leaf apex [22]. Francis was observed to have the longest leaves, followed by NA-7 and Banarasi, while Anand-2, Anand-1, Chakaiya, Krishna, and NA-10 had the shortest leaves. These variations can be important in determining the suitability of a particular cultivar for different purposes [23].

### 2.3. Physical Characteristics of Different Aonla Cultivars

Several cultivars, including Chakaiya, Banarasi, Francis, Kanchan, Krishna, Anand-2, Anand-1, NA-10, and NA-7, were described, along with their physical characteristics, which include traits unique to each cultivar, such as fruit weight, length, width, shape, fruit colour, flesh colour number of segments, fruit stalk, styler end, fruit stem end cavity, as well as pulp and juice percentage. Banarasi and Krishna cultivars had fruits with a triangular shape; Chakaiya, Francis, Kanchan, NA-7, and NA-10 cultivars had fruits of flattened round shape; while Anand-1 and Anand-2 cultivars had flattened oval shape fruit [24]. However, in terms of colour characteristics, Banarasi and Kanchan fruit had white to green; Chakaiya, Krishna, and Francis had a light green colour; Anand-2, NA-0 and NA-7 were yellowish green; and Anand-1 had a greenish yellow appearance [22]. Further NA-7, NA-10, Krishna, and Banarasi fruit stalks were observed to be short and thick, whereas Aanand-2, Aanand-1, Kanchan, Chakaiya, and Francis’s fruit stalks were observed to be short and thin. Chakaiya, Banarasi, Kanchan, Francis, Anand-2, and Anand-1 all had shallowed cavities of fruit stems, while Krishna, NA-7, and NA-10 had deep fruit stem end cavity [1]. Banarasi, Francis, Chakaiya, Kanchan, and NA 10 all had styler ends that were level; Krishna had styler ends that were observed to be prominent and less noticeable than Anand-2, Anand-1, and NA-7 [22]. All the types had six segments, although some, like Krishna and Kanchan, had as many as six or eight segments [25]. Except for Krishna, which had a light yellowish/green colour, other kinds had pale green coloured flesh. Fruit weight varied from 25.94 to 33.90 g, with Banarasi having the highest weight (33.90 g), followed by NA-7 (33.76 g), and Kanchan having the lowest weight (25.94 g) [21]. The fruit length varied from 3.07 to 3.82 cm; Kanchan had the longest fruits (3.82 cm), followed by Krishna (3.70 cm), Banarasi (3.73 cm), and Francis (3.07 cm), who had the shortest fruits, and Aanand-2 (3.12 cm) [26]. Fruit width between the cultivars ranged from 3.40 to 4.37 cm, with Banarasi having the widest fruit (4.37 cm), followed by Chakaiya and NA-7 (4.00 cm), and with Francis having the narrowest fruit (3.40 cm), Anand 2 having the next-smallest fruit (3.41 cm), and Anand-1 having the smallest fruit (3.40 cm) [16]. In NA-7 variety, estimated juice content was observed to be the greatest (64.45%), followed by Krishna (61.60%), and Chakaiya cultivars had the lowest content (40.00%), followed by Banarasi (41.34%). The pulp content ranged from 23.95 to 31.91 g/100 g of fruit, with Banarasi having the highest pulp content (31.91 g), with NA 7 (31.79 g), and Krishna (31.51 g), and Kanchan having the lowest pulp content (23.95 g), followed by Anand-1 (26.48 g) and Anand-2 (24.59 g) [27]. The structural characteristics of different aonla cultivars are presented in Table 1.

### 2.4. Chemical Characteristics of Different Aonla Fruit Cultivars

**Figure 2:**
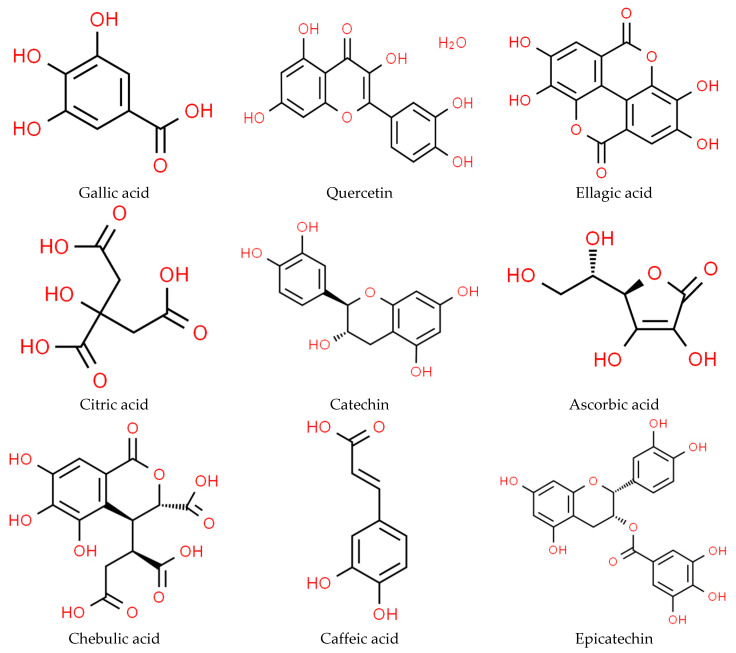
Chemical structure of different component’s present in aonla.

The different aonla cultivars such as Chakaiya, Banarasi, Kanchan, NA-7, and Desi and their chemical characteristics, which include features specific to each cultivar such as total soluble solids (TSS), acidity, pH, reducing sugar, non-reducing sugar, total sugar, starch, and ascorbic acid, were described. Desi variety and Chakaiya were observed to have the highest astringency, while NA 7 had the lowest astringency, although the other kinds were observed to be quite astringent. Kanchan had the lowest result (2.03%), followed by Krishna (2.16%) and Banarasi (2.21%), with their acidity range being 2.03–2.21% [1]. Fruits from the Banarasi and Kanchan varieties were of lower acidity than those from the Desi varieties [30]. The content of ascorbic acid in aonla ranges from 400 to 900 mg/100 g on a fresh weight basis. In contrast to other measures, pH and TSS values showed less volatility [28]. The total sugar content ranged from 28.01 to 36.91%. Pectin content ranged from 2.25 to 11.19%. Pectin content was observed to be the highest in Desi, Chakaiya, and NA-7. Starch content varied from 16.07 to 29.23% [31]. The fruits with the highest value of starch were observed from the Chakaiya variety, followed by those from the Desi variety, while the fruits with the lowest starch content were observed in the Banarasi variety [32]. The estimated ascorbic acid concentrations of all the cultivars varied from 334.12 to 453.20 mg/100 g. NA-7 (453.20 mg/100 g) and Kanchan (427.27 mg/100 g) had the highest concentrations, whereas Banarasi (334.12 mg/100 g) and Krishna (352.45 mg/100 g) had the lowest concentrations [33]. The highest level of total soluble solids was observed in NA-7 (11.50 °Brix), while the lowest level was found in Banarasi (8 °Brix) [21]. The chemical structures of different components present in aonla fruit are presented in Figure 2.

### 2.5. Mineral Characteristics of Different Aonla Cultivars

The different aonla cultivars like Chakaiya, Banarasi, Kanchan, NA-7 and Desi and, as well as their mineral characteristics, including respective mineral contents, sodium, calcium, zinc, iron, and potassium, were described. Potassium, calcium, and iron concentrations are observed in larger quantities and are regarded as macroelements, but zinc and sodium concentrations are observed in smaller amounts and classified as microelements. Sodium content was as follows: Chakaiya (69.37 ppm), Banarasi (53.71 ppm), Kanchan (71.51 ppm), NA-7 (64.49 ppm), and Desi (58.72 ppm) [28]. Zinc content was as follows: Chakaiya (65.56 ppm), Banarasi (45.66 ppm), Kanchan (50.48 ppm), NA-7 (53.09 ppm), and Desi (49.65 ppm) [30]. Iron content was as follows: Chakaiya was (3.10 mg), Banarasi (1.77 mg), Kanchan (2.46 mg), NA-7 (2.73 mg), and Desi (1.86 mg/100 g). Potassium content was as follows: Chakaiya is (62.20), Banarasi (58.22 mg), Kanchan (64.36 mg), NA-7 (63.90 mg), and Desi (43.67 mg/100 g) [1]. The calcium and iron contents of the fruit samples ranged from 17.84 to 28.40 mg/100 g and 1.77 to 3.10 mg/100 g, respectively [34]. As reported by the author, the Chakaiya variety displayed the greatest value for zinc and iron concentrations, whereas the Banarasi variety was observed to have the lowest zinc and iron concentrations. The zinc concentration was observed to be between 45.66 and 65.56 ppm [35]. The Nutritional characteristics of different aonla cultivars are presented in Table 2.

### 2.6. Total Polyphenol and Antioxidant Activity of Different Aonla Fruit Cultivars

Total polyphenol and antioxidant activity of different aonla fruit cultivars (Chakaiya, Banarasi, Kanchan, NA-7, and Desi) was evaluated by many authors using different solvents, such as methanol, ethanol, and ethyl acetate. Ethanol and ethyl acetate extract, followed by methanol extract, yielded the highest number of total phytochemicals. The concentration of ascorbic acid in different aonla extracts, in terms of mg/100 g, varied from 29.89 to 375.97. The ethanol extract of the Desi cultivar had the greatest ascorbic acid content (375.97 mg/100 g). According to Nambiar et al. [40], the methanol extract of aonla pulp contains 220.183 mg/100 g, while aqueous aonla extract contains 32.5 mg/g more ascorbic acid [41]. Comparing all phenolic components in all extracts, the quantity of quercetin (a flavonoid) was observed to be the lowest by different authors. However, among phenolics, gallic acid concentrations in aonla extract vary from 21 to 233.49 mg/100 g, while bethyl acetate extract of aonla contains 5.847% gallic acid (GA). Further, among the different cultivars, ellagic acid was a major phenolic compound and was present in aonla extracts in higher concentrations of 33.98 to 349.51 mg/100 g. Acetone was the most effective solvent for extracting GA, proceeded by ethanol, methanol, and water, according to Bhattacherjee et al. [42]; depending on the aonla type, ethyl gallate concentrations in various aonla extracts ranged from 0.24 to 41.96 mg/100 g. The ethanol extract of desi variety had the highest concentration of ethyl gallate. Higher amount of phytoconstituent were reported in the methanolic extracts of fresh aonla, such as ascorbic acid (2.15%), ethyl gallate (0.14), gallic acid (0.44%), and ellagic acid (0.15%). Further, aonla extracts also have higher flavonoid concentrations (quercetin) that vary from 0.24 to 1.94 mg/100 g. However, the methanol extract of NA-7 has the highest concentration of flavonoids and quercetin, followed by Chakaiya Desi, Banarasi, and Kanchan. Kaur et al. [43] discovered that the aqueous and ethanolic extracts of aonla had the higher antioxidant activity (84.3% and 86.8%, respectively). Among all cultivars, the Desi variety outperformed the other varieties in terms of antioxidant activity, followed by Chakaiya, Banarasi, NA-7, and Kanchan. In different fresh fruit extracts, antioxidant activities ranged from 35.2% to 85.6%, depending on the variety [44]. Moreover, a similar study by Kumar et al. [45] on the ethanol extract of aonla recorded a lower range of quercetin of 0.87–0.93 mg per 100 g. Additionally, the presence of these bioactive compounds also contributes to a higher antioxidant activity.

### 2.7. Antimicrobial Activity of Different Aonla Fruit Cultivars

Antimicrobial activity of different aonla cultivars, like Chakaiya, Banarasi, Kanchan, NA-7, and Desi, including their inhibition zones of *E. coli*, *Salmonella typhi*, *Candida albicans*, *Staphylococcus aureus* by using methanol, ethanol, and ethyl acetate, were described. The inhibition zones of *Salmonella typhi* and *Candida albicans* were reported to be 33.8, 10.9, 10.8, and 22.7 mm, respectively [45]. Without considering extracts, the Desi variety had the strongest antibacterial potential among the varieties, followed by Kanchan, Chakaiya, Banarasi, and NA-7. Different levels of sensitivity to infections could explain the differences in potential activity [46]. Maximum anti-microbiological action against *S. typhi* was discovered in the methanol extract of the Desi variety (4.30 mm), whereas the ethanol extract of the Chakaiya (1.42 mm) had the lowest anti-microbiological effect [23]. While the methanolic extract of the Chakaiya variety had the lowest antibacterial activity against *C. albicans* (1.12 mm) and *S. aureus* (1.91 mm), the ethanolic extract of the Desi variety demonstrated the highest antimicrobial activity against *S. aureus* (5.88 mm), *E. coli* (2.55 mm), and *C. albicans* 3.66 mm [47]. The extract of ethyl acetate from the cultivar Chakaiya was observed to have the lowest antibacterial activity (1.20 mm) against *E. coli* [11]. The total polyphenol, antioxidant activity, and antimicrobial activity of different aonla cultivars are presented in Table 3.

## 3. Clinical Studies on Different Extracts of Aonla

**Figure 3:**
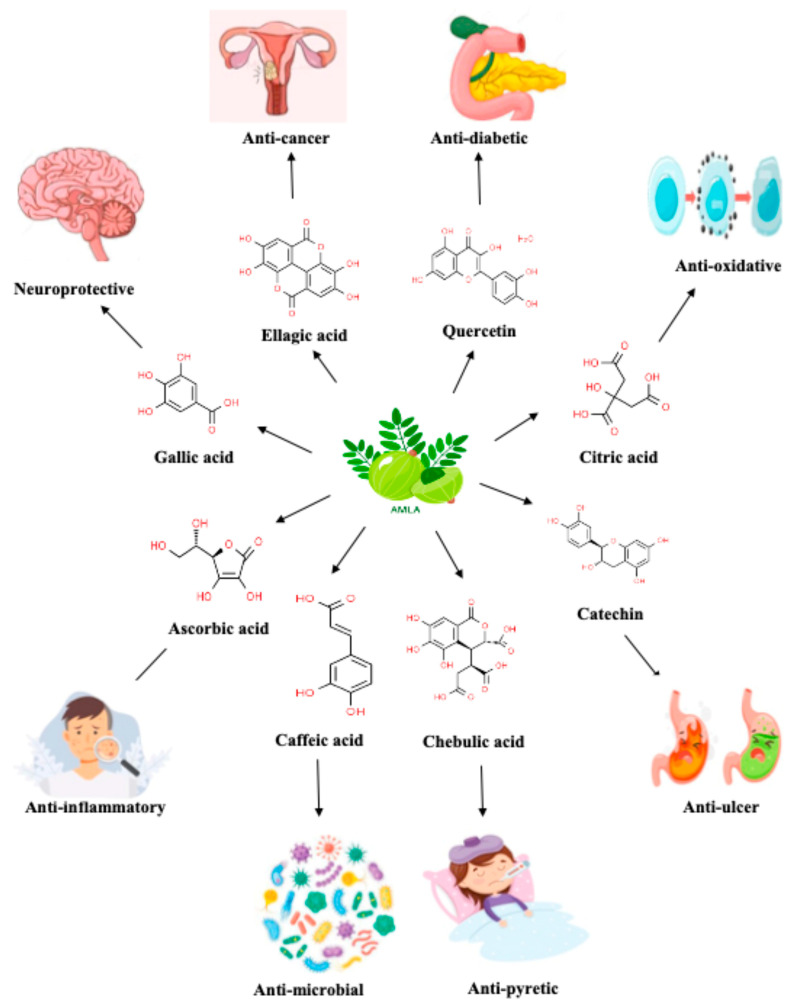
Medicinal properties of aonla.

Aonla contains different polyphenols, like gallic acid, ellagic acid, ascorbic acid, quercetin, citric acid, epicatechin, caffeic acid, chebulic acid, chebulanin acid, and chebulagic acid, which play important roles in performing antioxidant, analgesic, antipyretic, ulcerogenic, antidiabetic, gastrointestinal, anti-inflammatory, antimutagenic, anticancer, anti-carcinogenic, antitumor, antipathogenic, neuroprotective, and free radical scavenging activities that are helpful in curing numerous diseases, as mentioned in the data given below by various authors in their clinical studies. The various medicinal properties of aonla are presented in Figure 3.

### 3.1. Human Beings

Mahata et al. [49] reported the effects of *Phyllanthus emblica* on the treatment of cervical cancer in humans. Cervical cancer cells were exposed to PE (*P. emblica* fruit extract) doses of 10, 30, 100, and 300 g/mL for various amounts of time. They reported that *P. emblica* conveys its anticancer properties by inhibiting AP-1 and stops the expression of chromosomal abnormalities responsible for the progression and onset of ovarian cancer, suggesting the great potential of this compound in the treatment of cervical cancers caused by HPV (human papillomavirus). Kunchana et al. [50] reported the effects of *Phyllanthus emblica* fruit extract in curing the deadliest form of skin cancer, melanoma, which is associated with ultraviolet B (UVB) exposure. High-performance liquid chromatography was used to investigate the phytochemical components of PE (HPLC) (ascorbic acid, gallic acid, ellagic acid, quercetin, and chlorogenic acid) and UVB exposure in HaCaT cells (40 mJ per cm^2^). Further, the author concluded that PE considerably reduced apoptosis and elevated cell viability (MTT test) (Hoechst staining). The inflammatory reaction to UVB exposure was reduced via PE. Alsahli et al. [51] reported the protective effects of *Azadiractha indica* and *Phyllanthus emblica* against inflammation, glycation, and other oxidative stress-related issues. To test its in vitro anti-inflammatory and anti-glycating potentials, *Phyllanthus emblica* fruit pulp ethanol extracts were prepared. Both extracts (600 g/mL) greatly slowed down the development of AGEs, aggregation, structural alterations, and browning. Further, they concluded that both extracts were excellent antioxidants and could be used to treat inflammation and prevent the breakdown of biomolecules caused by glycation and oxidative stress. Usharani et al. [52] reported that an aqueous extract of fruits from *Phyllanthus emblica* given twice daily at 500 mg and 250 mg dosages would affect endothelial dysfunction (ED), systemic inflammation, oxidative stress, and lipid profile in subjects with MetS. The aqueous extract significantly improved endothelial function, systemic inflammation, oxidative stress, and lipid profile at both tested dosages, but particularly at 500 mg twice daily. This medication may be used in conjunction with conventional treatment to manage metabolic syndrome. Chaikul et al. [53] evaluated the potency of amla branches as anti-aging treatments. The survival of cells was given 0.1 mg/mL of PE extract. A dark brown powder called amla branch extract included many phenolic acids, primarily ferulic and sinapic acids. In vitro tests showed that the extract has strong antioxidant and tyrosinase-inhibitory properties, and cellular tests using the extract at 0.1 mg/mL demonstrated that it suppresses melanin. According to the conclusions of this study, amla branches are a rich source of bioactive compounds and may be utilized as an ingredient in cosmetics to delay the appearance of wrinkles.

### 3.2. Animals

#### 3.2.1. Rats

Nain et al. [54] evaluated the hypoglycaemic and antioxidant effects of *Emblica officinalis* Gaertn. leaf hydro-methanolic (20:80) extract. By administering oral treatment of the HMELEO at concentrations of 100, 200, 300, and 400 mg/kg bodyweight for 45 days daily in comparison to rats with diabetes, a significant decline in increasing blood glucose and an increase in insulin level were observed. According to the results, the groups treated with *Emblica officials* Gaertn. hydro-methanolic extract rather than glibenclamide may be more able to restore the damaged antioxidant state in streptozotocin-induced diabetes in a dose-dependent manner. However, in another study, various authors have reported the effects of different forms of Amalaki (group-A dosage level—1 g), Guduchi (group-B dosage level—1 g), and both capsules (group-C dosage level—500 g of each) on 30 individuals (14 men and 16 women; aged 25 to 60) with premature aging [55]. They further reported that the dried fruit rind of Amalaki and the stem of Guduchi were helpful in preventing premature aging in rats. Chatterjee et al. [56] conducted a study to observe the potential of gallic acid-enriched ethanolic extract (GAE) of *Phyllanthus emblica* fruits in treating mice with stomach ulcers brought on by indomethacin. The acute ulceration effectively healed after three days of treatment with omeprazole (3 mg/kg) and GAE (5 mg/kg) per day, and GAE was able to counteract the pro-inflammatory effects of indomethacin alterations in the specified biochemical markers. GAE exhibits anti-ulcerogenic qualities. By increasing the i-NOS/e-NOS ratio and promoting PGE2 production, GAE therapy speeds up ulcer healing. Asmilia et al. [57] revealed that the ethanol extract from Malacca leaves (*Phyllanthus emblica*) might help mice with oedema brought on by carrageenan at a level of 1%. For this investigation, 25 mice were selected and split into 5 groups. The extract from Malacca leaves and piroxicam was not provided to the mice in group 1. The rats in group 2 were treated with a suspension of piroxicam 20 mg. Malacca leaf ethanol extract was administered to mice in groups 3, 4, and 5 at 100, 200, and 300 mg/kg body weight dosage. Further, they reported that an ethanol extract of Malacca leaves could reduce oedema in male rats. Ojha et al. [58] reported that *E. officinalis* protects rats against the cardiotoxicity caused by isoproterenol (ISP). Rats were administered ISP subcutaneous injections (85 mg/kg, administered once per 24 h) and *E. officinalis* (100, 250, and 500 mg/kg, p.o.) for 30 days. Pre-treatment with *E. officinalis* significantly reduced lipid peroxidation and preserved myocyte-injury-specific marker enzymes and antioxidants. Additionally, the protective benefits of *E. officinalis* were validated by the histological recovery of myocardial tissue. Further, the findings showed that *E. officinalis* has cardioprotective potential. This potential is attributed to the plant’s powerful free radical scavenging and antioxidant activity. Sharma et al. [59] observed the preventive effects of triphala (a mixture of fruit powders from *Emblica officinalis*, *Terminalia belerica,* and *Terminalia chebula)* against (DMH)-induced endoplasmic reticulum stress (ER stress) in rats. After acquiring an oral dose of 3 mg/kg bodyweight in drinking water for 5 weeks, the serum levels of the enzymes glutamate oxaloacetate transaminase (SGOT), alkaline phosphatase (ALP), glutamate pyruvate transaminase (SGPT), and total bilirubin significantly increased, indicating biliary dysfunction and liver damage in mice. Further, they reported that the protective effects of triphala may be brought about by its active components’ stimulation of hepatic regeneration, which protects against the harm caused by the removal of electrophiles created by the metabolism of DMl, like their neutralization and elimination of alkyl-free radicals. Vishala et al. [60] assesses the antioxidant and nootropic impacts of *Emblica officinalis* on Alzheimer’s disease (AD) of Wistar rats brought on by aluminium. For 28 days, 4.2 mg/kg of aluminium chloride was administered intra peritoneally for rats to develop Alzheimer’s disease. In preventive experiments, aluminium chloride was administered together with plain amla powder (500 mg/kg) and unfortified amla powder (300 mg/kg). Rats that that developed Alzheimer’s disease were administered enriched amla powder (500 mg/kg) in curative research. The administration of plain and fortified amla fruit powder reduced malondialdehyde (MDA) levels and increased superoxide dismutase (SOD) and catalase levels. Neuroprotective and antioxidant efficacy against AD that is caused by aluminium chloride is made possible with *E. officinalis*. Tasanarong et al. [61] reported the effects of *Phyllanthus emblica* (PE) extract in preventing CI-AK. Male Sprague–Dawley mice were separated into eight groups before the introduction of CI-AKI and fed either water (control) or PE extract (125. 250 or 500 mg/kg daily) for five days. PE extract effectively reduced the severity of pathological damage and protected renal function. PE extract is shown to have a protective effect against CI-AKI. Kumar et al. [62] reported that *P. emblica* therapy prevented tumour number, incidence, and volume at different organ locations in several mouse cancer model studies. Antioxidants, changes to Phase I and II enzymes, cell cycle management, anti-inflammatory effects, and the mutation of oncogenic signalling genes are just a few of the various mechanisms that gooseberries use to inhibit cancer. Arun et al. [63] reported the effects of extract from aqueous leaves of PE on the protein levels of testicular dysfunction and metabolic changes in rats during long-term stress. Four groups of adult male rats were created: a chronic stress (CS) group, a control group, and two CS groups that received PE extract at various levels (50 or 100 mg/kg bodyweight). According to the research, PE (50 mg/kg BW) dramatically reduced MDA levels, sperm head deformities, corticosterone levels, and acrosome-reacted sperm in CS mice while considerably increasing sperm concentration and testosterone levels. PE was also seen to reduce histopathology of the testicles in the CS rats at both dosages.

#### 3.2.2. Fish

Wu et al. [64] examined phytomedicines for their potential impacts on atherosclerosis development in vivo using fluorescent imaging in Zebra fish. *P. emblica* fruit ethyl-acetate extract (E-EA) was shown to be very successful in minimizing vascular monocyte infiltration. The E-EA also decreased the accumulation of vascular cholesterol in Zebrafish fed a high-fat diet. In vitro oxidized LDL, the stimulation of macrophages resulted in a net accumulation of cholesterol that E-EA inhibited. Further, they reported that *P. emblica* fruit might inhibit foam cell formation and macrophage recruitment, inhibiting vascular lipid deposition and curing atherosclerosis in zebrafish. Doan et al. [65] reported the effects of amla fruit extract (AFE) (*Phyllanthus emblica*) on Nile tilapia (*Oreochromis niloticus*) development, serum and skin mucosal immunity, and disease resistance against *Streptococcus agalactiae*. For 8 weeks, 300 fish (10.48–0.56 g fish each fish) were administered a base meal supplemented with 0 mg, 5 mg, 10 mg, 20 mg, and 40 mg kg^−1^ AFE. There were 20 fish per tank, distributed among 15 tanks. A significant improvement in growth metrics was seen when AFE was added for 8 weeks, with the 20 mg per kg AFE group showing the fastest growth rate. In conclusion, nutritional supplementation with 20 mg AFE kg-1 may be employed in Nile tilapia aquaculture as an immune stimulant and growth promoter.

#### 3.2.3. Cow

Patel et al. [66] reported the effects of *Tinospora cordifolia* and *Emblica officinalis* supplementation on growth performance, immunological status, and hormonal condition in murrah calf buffalos. Six nonsupplemented calves at 14 days of age were used as the control group, whereas six calves in the treatment group received a combination of *Tinospora cordifolia* and *Emblica officinalis* (1:2) at the rate of 450 mg once a day for 28 days, based on bodyweight. Further, they reported that *Tinospora cordifolia* and *Emblica officinalis* have medicinal benefits, and the combination of *Tinospora cordifolia* and *Emblica officinalis* has immunogenic properties which reduce calf mortality by producing a generalized increase in resilience to different environmental conditions. Tilahun et al. [67] reported the results of feeding lactating dairy cows three levels of a new feedstock made from fresh amla fruit. In a repeated crossover design, eighth dairy cows ruminal cannulated in the middle of lactation were randomly divided into two treatment groups. The beginning of period 1 was immediately followed by a 14-day adaptation phase. During this period, the first four cows had a control diet, while the other four cows received FAF supplements at successively increasing dosages (200, 400, and 600 g/day), spaced by 14-day intervals. PE boosts butter quality, protein efficiency, antioxidant capacity, and ideal milk fatty acid profiles, which reduces cow arthritis.

#### 3.2.4. Plants

Pandey et al. [68] reported *Phyllanthus emblica* aqueous extract to reduce chromium toxicity’s harmful consequences in rice seedling development, reactive oxygen species (ROS) production, Cr absorption, antioxidative enzyme activities, and lipid peroxidation. Rice seedlings were grown hydroponically for 4–8 days in either 100 M Cr (K2Cr2O7) or Yoshida nutritional medium with Cr + *P. emblica* aqueous extract (5 mg/mL). According to the investigation, an aqueous extract of *P. Emblica* significantly reduces the negative effects of chromium toxicity in rice seedlings by inhibiting Cr absorption, regulating the activities of antioxidative enzymes, and lowering oxidative stress. Clinical studies of different aonla cultivars are mentioned in Table 4.

## 4. Application of Aonla in Value-Added Products

### 4.1. Beverages

Gaikwad et al. [69] reported that herbal RTS prepared by aonla ginger using artificial sweeteners like aspartame was observed to be rich in vitamins and was found with an ascorbic acid content of 180 mg/100 g. Moreover, it is highly appreciated due to the characteristics of customer-related habits.

### 4.2. Dried Products

Dutta et al. [70] reported that aonla shreds, even in dry form, contain a significant amount of vitamin C. Leuco-anthocyanins or polyphenols are credited with preserving ascorbic acid in dried aonla products. Mishra et al. [33] reported that aonla powder prepared by freeze drying retained iron, phosphorus, calcium, iron, ascorbic acid, and colour better than spray drying. Kore et al. [71] reported that aonla candy prepared with aonla and sugar is highly preferable among fruit candy due to various characteristics such as low volume, high storage stability, better nutritional profile, and overall acceptability. Additionally, these types of candy were easy to prepare and ready-to-eat snacks. Aonla pills prepared with aonla pulp, ground cumin, ginger, and salt are incredibly tasty, easy to digest, and full of vitamin C.

### 4.3. Chutney, Sauce, Pickle, and Preserves

Dutta et al. [70] reported that aonla chutney typically has a mellow flavour and is hot, sweet, smooth, and spicy. The addition of raisins and dried fruits can improve the flavour and nutritional value of aonla chutney. Kore et al. [71] reported that aonla sauce prepared with aonla, tomato pulp, sugar, salt, onion, garlic, ginger, red chilies, and spices was observed to have a long shelf life and is still quite suitable even after more than nine months of storage. Pavitra et al. [72] reported that aonla pickles prepared from aonla, salt, turmeric, red chili powder, fenugreek, clove, and oil is rich in vitamin C, and they also prevent the formation of ulcers. Kore et al. [71] reported that aonla preserves were observed to have a positive impact of cleansing blood in addition to assisting in lowering cholesterol levels and enhancing vision, and they also observed to have an extended shelf life.

### 4.4. Ayurvedic Products

Athawale et al. [73] reported that chyawanprash prepared from aonla and other herbal ingredients aids in regenerating and activating body cells. The author also reported that consuming a spoonful of chyawanprash everyday may function as a powerful immune booster, which also tones muscles and promotes the synthesis of white blood cells, hemoglobin, and the spleen. It is used to treat issues with fertility as well as respiratory health. It restores the metabolic processes and treats abnormalities like gas and coughing. Jat et al. [8] reported that the ashokrishtaan ayurvedic tonic is prepared from the ashoka tree is combined with amla and other medical plants like haritaki, dhataki, jeera, musta, mango seeds, and jaggery, with it being mostly used by women to maintain a hormonal balance. It helps increase fertility and is beneficial for women who experience emotional and hormonal ups and downs. It is beneficial for treating pelvic inflammatory illness, reducing menstrual cramps, preventing osteoporosis and the loss of minerals from the bones, promoting digestion, and enhancing stamina due to its high anti-inflammatory and analgesic content.

### 4.5. Other Products

Bishnoi et al. [74] reported that aonla ladoo prepared from the roots of satavari and aonla is nutritious and has a longer shelf life during storage. During three months of storage, it was also observed to have an increasing TSS, reduced total sugars, and decreasing trend in titratable acidity, tannin, and ash. The various application of aonla in value-added products is presented in Table 5.

## 5. Conclusions

The thorough analysis of the literature on aonla, or Indian gooseberry, concludes that this fruit has exceptional medicinal and nutritional potential. Aonla fruit’s potential as a functional food can be claimed by its high nutritional, phytochemical, antioxidant, antimicrobial, and mineral composition. Aonla’s diverse range of health benefits, including its ability to reduce fever, reduce inflammation, oxidative stress, age, prevent ulcers, chemoprotection, neuroprotection, glycaemic control, and immune system boost, make it a powerful natural remedy for a variety of health conditions. It is imperative that clinical trials should be the primary focus of future research directions to substantiate the traditional applications and documented health benefits of aonla. All these factors lead to aonla fruit being a potential emerging raw material for innovative pharmaceuticals and nutraceutical development. Prioritizing research into the creation of low-cost dietary supplements based on aonla can help to maximize the benefits of its entire range of health-promoting components. Lastly, research on environmentally friendly harvesting and processing techniques will be essential for guaranteeing that the advantages of aonla can be efficiently and extensively disseminated, improving the health of individuals worldwide.

## Figures and Tables

**Table 1:** Structural Characteristics of aonla fruit cultivars.

**Morphological Characteristics of Aonla Cultivars**										
**Varieties/Characteristic**	**Tree Height (m)**	**Tree Habit**	**Tree Form**	**Leaf Size (cm)**	**Leaf Form**	**Leaf Width (cm)**	**Leaf Apex**	**Foliage**	**Inflorescence Colour**	**References**
Chakaiya	3.78	Upright	Spreading	1.24	Oblong	0.30	Acute	Sparse	Pinkish green	[1,20,21,22,23]
Banarasi	3.90	Upright	Spreading	1.29	Oblong	0.37	Obtuse	Sparse	Deep green	
Francis	4.21	Tall	Dropping	1.47	Oval oblong	0.32	Obtuse	Dense	Yellowish green	
Kanchan	4.59	Tall	Spreading	1.29	Oval oblong	0.32	Acute	Sparse	Pinkish green	
Krishna	3.86	Upright	Spreading	1.29	Oblong	0.31	Obtuse	Sparse	Deep green	
Anand-2	5.67	Tall	Upright	1.25	Oblong	0.23	Obtuse	Sparse	Light green to pinkish	
Anand-1	5.52	Tall	Upright	1.27	Oblong	0.26	Obtuse	Sparse	Green to light pink	
NA-10	3.89	Semi tall	Semi spreading	1.28	Oblong	0.30	Obtuse	Dense	Green to light pink	
NA-7	4.03	Tall	Semi-Spreading	1.40	Elliptical	0.30	Obtuse	Dense	Green to light pink	
**Physical and structural characteristics of aonla fruit cultivars**										
**Varieties/Characteristics**	**Fruit Weight (gm)**	**Fruit Length (cm)**	**Fruit Width (cm)**	**Fruit Shape**	**Fruit Colour**	**No of Segments**	**Styler End**	**Pulp (g/100 g of Fruit)**	**Juice (%)**	**References**
Chakaiya	30.66	3.35	4.00	Round Flattened	Pale green	6	Levelled	28.64	40.00	[1,16,21,22,24,25,27,28,29]
Banarasi	33.90	3.73	4.37	Triangular	Whitish green	6	Levelled	31.91	41.34	
Francis	30.41	3.07	3.40	Round Flattened	Whitish green	6	Levelled	28.33	52.12	
Kanchan	25.94	3.82	3.99	Round Flattened	Whitish green	6–8	Levelled	23.95	48.12	
Krishna	33.56	3.70	3.93	Triangular	Pale green	6–8	Prominent	31.51	61.60	
Anand-2	26.63	3.12	3.41	Flattened oval	Yellowish green	6	Less prominent	26.48	46.15	
Anand-1	28.53	3.10	3.45	Flattened oval	Greenish yellow	6	Less prominent	24.59	44.52	
NA-10	31.45	3.39	3.98	Flattened Round	Yellowish green	6	Levelled	29.46	58.15	
NA-7	33.76	3.62	4.00	Flattened Round	Yellowish green	6	Less prominent	31.79	64.45	
Desi	14.27	25.12	29.38	Round	Yellowish green	6	Less prominent	35.65	44.50	

**Table 2:** Nutritional characteristics of different aonla fruit cultivars.

Chemical Characteristics of Aonla Fruit Cultivars *										
**Fruit Cultivars/Characteristics**	**TSS (°Brix)**	**Acidity (%)**	**pH**	**Reducing Sugar (%)**	**Non-Reducing Sugar (%)**	**Total Sugar (%)**	**Starch (%)**	**Ascorbic Acid (mg/100 g)**	**Pectin (%)**	**References**
Chakaiya	10.6	2.10	2.88	22.17	14.73	36.91	29.23	399.76	2.98	[1,28,30,31,32,33,36,37,38,39]
Francis	9.70	2.02	-	2.65	1.38	4.03	-	571.15	-	
Banarasi	8.0	2.21	2.84	17.80	11.52	29.33	16.07	334.12	2.41	
Kanchan	10.2	2.03	2.82	18.19	10.65	28.84	16.42	427.27	2.28	
Krishna	10.62	2.19	-	-	-	7.61	-	494.00	-	
Anand-1	11.30	2.05	-	-	-	-	-	419.45	-	
Anand-2	12.70	2.34	1.97	7.60	-	7.91	-	120.95	-	
NA-7	11.50	2.05	2.82	17.58	15.26	32.85	18.49	453.20	2.25	
NA-10	14.19	2.63	-	-	-	9.49	-	473	-	
Desi	8.80	12.06	2.71	16.33	12.67	28.01	19.34	352.45	11.19	
Mineral characteristics of aonla fruit cultivars *										
**Fruit Cultivars/Characteristics**	**Sodium (ppm)**		**Calcium (mg/100 g)**		**Zinc (ppm)**		**Iron (mg/100 g)**		**Potassium (mg/100 g)**	**References**
Chakaiya	69.37		17.84		65.56		3.10		62.20	[1,28,30,34,35]
Banarasi	53.71		17.84		45.66		1.77		58.22	
Kanchan	71.51		24.73		50.48		2.46		64.36	
NA-7	64.49		21.385		53.09		2.73		63.90	
Desi	58.72		28.40		49.65		1.86		43.67	

* Fresh weight basis (FW).

**Table 3:** Total polyphenol, antioxidant activity, and antimicrobial activity of different aonla fruit cultivars.

**Total Polyphenol and Antioxidant Activity of Different Aonla Fruit Cultivars**								
**Varieties/Characteristics**	**Extract**	**Total Phenolics (mg/100 g)**	**Antioxidant Activity (%)**	**Ellagic Acid (mg/100 g)**	**Gallic Acid (mg/100 g)**	**Ascorbic Acid (mg/100 g)**	**Quercetin (mg/100 g)**	**References**
Chakaiya	Methanol	375.98	66.7	155.16	83.74	136.48	0.27	[40,41,42,43,44,45]
Banarasi		391.42	74.4	124.11	87.68	178.96	0.34	
Kanchan		493.7	69.6	167.41	199.05	126.42	0.55	
NA-7		477.2	75.2	113.35	154.77	206.75	1.94	
Desi		752.65	75.2	349.51	233.49	167.19	1.48	
Chakaiya	Ethanol	324.62	66.0	50.22	43.83	228.25	0.56	
Banarasi		336.72	70.6	43.61	64.90	224.82	0.47	
Kanchan		378.07	74.3	33.98	46.72	293.51	0.87	
NA-7		436.33	71.9	38.70	48.46	342.96	0.24	
Desi		533.29	85.6	61.62	52.55	375.97	1.19	
Chakaiya	Ethyl acetate	158.47	35.2	92.44	83.16	65.82	0.34	
Banarasi		164.3	39.8	58.57	67.57	36.99	0.64	
Kanchan		149.35	36.9	41.84	53.40	53.55	0.32	
NA-7		104.46	40.3	44.52	29.30	29.89	0.49	
Desi		299.85	46.5	92.44	83.16	122.47	0.74	
**Antimicrobial activity of different aonla fruit cultivars**								
**Variety/Characteristics**	**Extract**	***E. coli***		***Salmonella typhi***		***Candida albicans***	***Staphylococcus aureus***	**References**
Chakaiya	Methanol	1.33		3.35		1.12	1.91	[11,23,45,47,48]
Banarasi		1.61		3.75		1.36	4.57	
Kanchan		1.38		3.74		1.13	4.16	
NA-7		1.70		3.91		1.46	5.16	
Desi		1.85		4.30		1.50	5.22	
Chakaiya	Ethanol	2.07		1.42		1.53	5.88	
Banarasi		2.33		2.41		2.86	3.72	
Kanchan		2.10		2.27		2.66	3.49	
NA-7		2.51		2.99		3.25	5.29	
Desi		2.55		3.10		3.66	5.88	
Chakaiya	Ethyl acetate	1.20		2.14		1.39	2.74	
Banarasi		1.34		2.33		1.65	4.26	
Kanchan		1.30		2.17		1.57	3.14	
NA-7		1.48		2.60		1.80	4.52	
Desi		1.52		2.81		1.81	5.34	

**Table 4:** Clinical studies on different aonla extracts.

Type	Disease	Treatment	Description	Result	References
Humans	Cervical cancer	PE concentrations (10, 30, 100, 300 g/mL) with varied time.	PE prevented the formation of cervical cancer by inhibiting AP-1.	PE shows anti-AP-1 and anti-HPV activity which cures cervical cancer in human.	[49]
	Skin Cancer	Solution of PE 10, 50, and 100 μg/m.	PE assessment against UVB-induced keratinocyte inflammation and apoptosis was studied.	PE reduced inflammation and shielded HaCaT cells from UVB-induced oxidative damage.	[50]
	Oxidative stress	PE 600 g/mL.	Therapeutic potential of PE was investigated.	Antioxidants of PE extract treats inflammation by preventing the oxidative stress.	[51]
	Endothelial dysfunction	Dose of 250 and 500 mg of *Phyllanthus emblica* twice daily.	Effects of PE on ED, oxidative stress, systemic inflammation, and lipid profile of patients were investigated.	PE enhanced endothelial function considerable reduction was seen in RI, GSH, and ED.	[52]
	Aging in humans	The resilience of cells given 0.1 mg/mL PE extract treatment.	Effects of amla branch extract in delaying aging process was investigated.	Amla shows anti-aging properties, including skin lightening, wrinkle reduction, as well as improved elasticity and hydration.	[53]
Animals-Rat	Diabetes in rats	HMELEO dosage 100, 200, 300, and 400 mg/kg bodyweight orally for 45 days.	Diabetes caused by streptozotocin shows enhanced oxidative stress in rats.	EO extract shows hypoglycaemic effects, antidiabetic effect and decrease in oxidative stress.	[54]
	Premature ageing	Amlaki (capsule containing 500 mg of amla powder) 1 g/day once daily for 3 months.	Study conducted on 30 individuals with stress-related premature ageing.	Amalaki capsule slow down the effects of stress-related premature ageing.	[55]
	Gastric ulcer	Omeprazole/day dosage of 3 mg/kg and GAE dosage of 5 mg/kg per day for 3 days.	Indomethacin 18 mg/kg; administration of single dose resulted in stomach ulceration.	GAE shows anti-ulcerogenic property and speeds up the healing of ulcers.	[56]
	Oedema in mice	Malacca leaf ethanol extract dosage 300, 200, and 100 mg/kg b.w.	Carrageenan (1%), piroxicam suspension (20 mg), and Malacca leaf used to cause inflammation.	Malacca leaf ethanol extract cures oedema in mice.	[57]
	Cardiotoxicity in rats	ISP injections (85 mg/kg, given once per 24 h) and *E. officinalis* (100, 250 and 500 mg/kg, p.o.).	Cardiotoxicity in rats is induced by isoproterenol.	Antioxidant activity of E.O. is attributable to its cardio-protective potential, showing improvements in hemodynamic contractile function.	[58]
	Carcinogenic damage	Triphala dosage 3 mg/kg b.w. orally for 5 weeks in drinking water.	1,2-dimethylhydrazinedihydrochloride causes ER stress in mouse liver (DMH).	Triphala protects liver from early neoplastic changes brought on by DMH and ER stress.	[59]
	Alzheimer in rats	The aluminium chloride was administered along with the unprocessed amla powder 500 mg/kg and unfortified amla powder 300 mg/kg.	Disease was induced by injecting aluminium chloride intraperitoneally for 28 days with dosage of 4.2 mg/kg.	Antioxidant and radical-scavenging activities of amla shows neuroprotective behaviour in enhancing memory.	[60]
	Kidney injury	Dose of 125, 250, or 500 mg/ kg of PE extract daily.	CI-AKI resulted from the administration of intravenous iodinated contrast agents.	The antioxidant properties of aonla maintain renal function, and PE extract prevents CI-AKI.	[61]
	Tumour in mouse	*P. emblica* (10% sucrose, 18 days 100 mg/kg bodyweight/day.	PE lowers the expression of HIF-1 and endothelial cell antigen CD31 in mice xenograft tumours.	PE reduces HIF-1 expression and endothelial cell antigen CD31 in mice xenograft tumours.	[62]
	Chronic stress in rats	PE extract of 50 mg or 100 mg/kg bodyweight.	Effects of PE aqueous leaf extract on the changes in protein markers of stressed rats was investigated.	Modifications in testicular tyrosine-phosphorylated proteins; testicular injury in mice with CS is alleviated.	[63]
Fish	Atherosclerosis	E-EA was directly injected (10 g/mL final concentration) for 10 days.	Phytomedicines were examined for their influence on the in vivo development of atherosclerosis.	Inflammatory cells and vascular lipid were less prevalent in zebrafish larvae. E-EA prevents the onset of atherosclerosis.	[64]
	Inflammation in fish	Base diet supplemented with 0, 5, 10, 20, and 40 mg/kg AFE for 8 weeks.	Effects of PE on skin mucosal immunity and serum protection was investigated.	Dose of 10 mg AFE kg used as an immunostimulant and growth promoter in Nile tilapia aquaculture.	[65]
Cow	Immunomodulation of bovine calves	EO and *Tinospora cordifolia* (2:1) dosage 450 mg/ kg bodyweight once daily for 28 days orally.	The immunomodulation of bovine calves is the subject of research.	Combinations of EO and TC have immunogenic effects and reduce calf mortality.	[66]
	Atherogenicity	Fresh aonla fruit 200, 400 and 600 g/day for 14 days.	Milk antioxidant properties and milk fatty acid ratios were investigated.	PE increases butter quality, antioxidant activity, and protein efficiency and decreases atherogenicity in cows.	[67]
Plants-Rice	Chromium toxicity in rice	Dose of 100 μMCr (K2Cr_2_O7) or Cr + *P. emblica* aqueous extract for 4–8 days.	Effects of APE in minimizing the Cr toxicity on rice seedlings was investigated.	Oxidative stress was lowered with the inhibition of the uptake of Cr, and APE reduces the effects of Cr on rice seedlings.	[68]

PE—*Phyllanthus emblica*; EO—*Emblica officinalis*; CV—Chandraprabhavati; HMELEO—hydro-methanolic extract of *Emblica officinalis*; GAE—gallic acid-enriched ethanolic extract; ISP—isoproterenol; DMH—1,2-dimethyl hydrazine dihydrochloride; ER—endoplasmic reticulum; CI-AKI—contrast-induced acute kidney injury; HaCaT—high sensitivity of human epidermal keratinocytes.

**Table 5:** Application of aonla in value-added products.

Types	Value Added Product	Formulation	Use	References
Beverages	Aonla RTS	RTS is prepared using ginger and aonla.	Good source of vitamin C.	[69]
Dried products	Aonla shreds	Fruit grated into shreds and combined with 4% salt and dried to a moisture content of 15%.	High in vitamin C concentration.	[70]
	Aonla powder	Fruit juice was obtained and dried with spray drying method.	Excellent iron, phosphorus, calcium, and ascorbic acid retention.	[33]
	Aonla candy	Fruit that has been extracted, drained, and dried after being impregnated with sugar.	Higher acceptance, smaller volume, and enhanced nutritious content.	[71]
	Aonla pills	Prepared by drying aonla pulp and mixing it with powdered ginger, cumin, and salt.	Vitamin C-rich and digestive pills are incredibly tasty.	[70]
Chutney, sauce, pickle and preserve	Aonal chutney	Aonla chutney has delicious flavours that are fiery, sweet, smooth, spicy, and mellow.	It is tasty and nutritious.	[70]
	Aonla sauce	Prepared by using 50% aonla and 50% tomato.	Long shelf life.	[71]
	Aonla pickle	Fruit pieces were mixed with spices and oil for pickle production (1 kg aonla fruit, salt, turmeric, red chili powder, fenugreek, clove, and oil).	Rich in vitamin C, and it also prevents the formation of ulcers.	[72]
	Aonla preserve	Made from whole fruit or large pieces of fruits steeped in sugar until it is tender and translucent.	Beneficial for purifying the blood, lowering cholesterol levels, and improving vision.	[71]
Ayurvedic products	Chyawanprash	A mixture of ingredients including aonla, sesame oil, ghee, honey, and other ingredients.	Restores metabolic processes, treats abnormalities like gas and coughing, and actsas a powerful immunity booster.	[73]
	Ashokarishta	Amla is combined with ashoka tree decoction and other ingredients like mango seeds, jeera, haritaki, dhataki, and musta, with which a tonic is prepared.	Beneficial for treating pelvic inflammatory illness, reducing menstrual cramps, and for preventing osteoporosis and the loss of minerals from bones.	[8]
Other products	Aonlaladoo	Powdered satavari roots are used to prepare herbal aonla ladoo.	Nutritious and have more medicinal value.	[74]

## Data Availability

Not Applicable.
